# Distribution and favorable prognostic implication of genomic *EGFR* alterations in *IDH* ‐wildtype glioblastoma

**DOI:** 10.1002/cam4.4939

**Published:** 2022-06-13

**Authors:** Nayuta Higa, Toshiaki Akahane, Taiji Hamada, Hajime Yonezawa, Hiroyuki Uchida, Ryutaro Makino, Shoji Watanabe, Tomoko Takajo, Seiya Yokoyama, Mari Kirishima, Kei Matsuo, Shingo Fujio, Ryosuke Hanaya, Akihide Tanimoto, Koji Yoshimoto

**Affiliations:** ^1^ Department of Neurosurgery, Graduate School of Medical and Dental Sciences Kagoshima University Kagoshima Japan; ^2^ Department of Pathology, Graduate School of Medical and Dental Sciences Kagoshima University Kagoshima Japan; ^3^ Center for Human Genome and Gene Analysis Kagoshima University Hospital Kagoshima Japan; ^4^ Department of Neurosurgery, Graduate School of Medical Sciences Kyushu University Fukuoka Japan

**Keywords:** EGFR kinase domain, *EGFR* variant, *EGFRvIII*, glioblastoma

## Abstract

**Background:**

We aimed to evaluate the mutation profile, transcriptional variants, and prognostic impact of the epidermal growth factor receptor (*EGFR*) gene in isocitrate dehydrogenase (*IDH*)‐wildtype glioblastomas (GBMs).

**Methods:**

We sequenced *EGFR*, evaluated the *EGFR* splicing profile using a next‐generation sequencing oncopanel, and analyzed the outcomes in 138 grade IV *IDH*‐wildtype GBM cases.

**Results:**

*EGFR* mutations were observed in 10% of GBMs. A total of 23.9% of the GBMs showed *EGFR* amplification. Moreover, 25% of the *EGFR* mutations occurred in the kinase domain. Notably, *EGFR* alterations were a predictor of good prognosis (*p* = 0.035). GBM with *EGFR* alterations was associated with higher Karnofsky Performance Scale scores (*p* = 0.014) and lower Ki‐67 scores (*p* = 0.005) than GBM without *EGFR* alterations. *EGFRvIII* positivity was detected in 21% of *EGFR*‐amplified GBMs. We identified two other *EGFR* variants in GBM cases with deletions of exons 6–7 (Δe 6–7) and exons 2–14 (Δe 2–14). In one case, the initial *EGFRvIII* mutation transformed into an *EGFR* Δe 2–14 mutation during recurrence.

**Conclusions:**

We found that the *EGFR* gene profiles of GBM differ among cohorts and that *EGFR* alterations are good prognostic markers of overall survival in patients with *IDH*‐wildtype GBM. Additionally, we identified rare *EGFR* variants with longitudinal and temporal transformations of *EGFRvIII*.

## Graphical abstract

The frequency of epidermal growth factor receptor (*EGFR*) mutations was low but the frequency of *EGFR* mutations in the kinase domain was high in isocitrate dehydrogenase‐wildtype glioblastomas (GBMs) in our cohort. Notably, in this study, GBM with EGFR alterations had a better outcome than GBM without them.

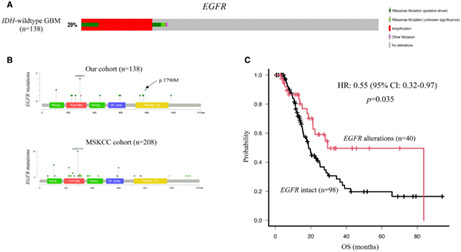

## 1 INTRODUCTION

The epidermal growth factor receptor (*EGFR*) gene is the most commonly amplified and overexpressed proto‐oncogene in glioblastoma (GBM). *EGFR* amplification is detectable by next‐generation sequencing (NGS) in approximately 40%–50% of GBM cases^1^, ^2^, ^3^ and 14.4%–26% of GBMs harbor *EGFR* mutations.^2^, ^3^ Recently, the Consortium to Inform Molecular and Practical Approaches to CNS Tumor Taxonomy (cIMPACT‐NOW) proposed a diagnostic entity of grade II–III isocitrate dehydrogenase (*IDH*)‐wildtype astrocytoma having either telomerase reverse transcriptase promoter (*TERTp*) mutation, *EGFR* amplification, or a combination of whole chromosome 7 gain and whole chromosome 10 loss (+7/−10) that behaves similar to GBM.^4^ The 2021 WHO classification accounts for these new insights by stipulating molecular criteria that allow for a diagnosis of GBM, CNS WHO grade IV, in *IDH*‐wildtype astrocytomas, even in the absence of high‐grade histopathologic features, when at least one of the following molecular features is present: *TERTp* mutation, *EGFR* amplification, or concurrent +7/−10.^5^ Owing to the molecular features defining this new tumor entity, *EGFR* may serve as a diagnostic and prognostic biomarker. Indeed, EGFR has garnered interest as a drug target in GBM because of the high frequency of *EGFR* alterations in this disease. However, GBMs respond poorly to EGFR inhibitors.^6^, ^7^ As *EGFR* mutations in GBM occur in the extracellular domain rather than the intracellular kinase domain, EGFR inhibitors that target the kinase domain are less effective.^7^

To date, the prognostic value of *EGFR* alterations in GBM remains controversial. Some studies have suggested that *EGFR* alterations correlate with a favorable outcome,^8^, ^9^, ^10^ while the association with worse prognosis has been reported in others.^11^ *EGFR* amplification and classical subtype were reported to be associated with poor response to bevacizumab.^12^ However, Hobbs et al. demonstrated that high *EGFR* amplification was associated with favorable prognosis and responded well to temozolomide.^9^

Splicing variants of *EGFRvII* (Δe 14–15 deletions), *EGFRvIII* (Δe 2–7 deletions), *EGFRvIV* (Δe 25–27 deletions), *EGFRvV* (Δe 25–28 deletions), and *EGFR* (Δe 12–13 deletions) have been reported in GBMs.^2^, ^3^, ^13^, ^14^ *EGFRvIII*, which is generated by the fusion of exons 1 and 8, is detectable in 50%–60% of *EGFR*‐amplified GBMs.^15^, ^16^ *EGFRvIII* lacks the extracellular ligand‐binding domain and constitutively activates the receptor tyrosine kinase domain that increases GBM cell proliferation, migration, and invasion.^17^, ^18^ *EGFRvIII* may serve as a tumor‐specific target for anti‐EGFRvIII immunotherapy, including antibody‐based approaches and vaccines, such as rindopepimut, which produces a survival signal when combined with bevacizumab in *EGFRvIII*‐positive recurrent GBMs.^19^ However, immunotherapy failed in a phase III clinical trial in newly diagnosed patients.^20^ This may be because of the frequent loss of *EGFRvIII* expression with standard chemoradiotherapy.

In this study, we collected a higher number of samples and performed NGS analysis using a custom gene panel that we reported recently.^21^ In addition, we analyzed the mutational distribution and transcriptional variants of *EGFR* and the clinical outcomes of grade 4 *IDH*‐wildtype GBM cases.

## 2 METHODS

### 2.1 *IDH* ‐wildtype GBM samples

One hundred and thirty‐eight formalin‐fixed paraffin‐embedded (FFPE) tumor tissue samples were selected from the Central Nervous System Tumor Tissue Bank at Kagoshima University Hospital. All tumors fulfilled the World Health Organization classification of 2021. The tumor series consisted of samples from 138 adult patients (over 20 years old) with *IDH*‐wildtype GBM in our database between February 2014 and September 2021. The study was approved by the Institutional Review Board of Kagoshima University (approval number: 180104) and complied with the tenets of the Declaration of Helsinki. Informed consent was obtained from all patients. Resected tumors were fixed with phosphate‐buffered 10% formalin within 24 h of sampling and routinely processed for paraffin embedding, followed by sectioning for hematoxylin and eosin staining. All tissues were histologically evaluated by board‐certified pathologists (M. K. and A. T.) to ensure an estimated tumor cell content of 30% or more. In all patients, when analyzing copy number (CN) variations, we sequenced leukocyte DNA for comparison against matched tumor DNA.

### 2.2 Treatments

We performed the removal of ≥ 90% of the tumor on 71 patients (51.4%) and the removal of < 90% of the tumor on 67 patients (48.6%). Additionally, we treated 134 GBM patients with temozolomide as per the Stupp protocol and also performed subsequent temozolomide maintenance treatments.^22^ However, four patients were not treated because of severe clinical conditions, such as advanced age or low Karnofsky Performance Scale (KPS) scores.

### 2.3 DNA extraction and quantification

For DNA preparation from FFPE samples, we used the Maxwell 16 FFPE Tissue LEV DNA Purification kit (Promega) according to the manufacturer’s instructions. Thereafter, the concentration of DNA was measured using a Qubit 3.0 Fluorometer dsDNA BR Assay kit (Life Technologies), and DNA quality was monitored using the QIAseq DNA QuantiMIZE kit (QIAGEN). The extracted DNA was diluted to a concentration of 5–10 ng/μL as a template, and PCR was performed using the QIAseq DNA QuantiMIZE kit.

### 2.4 Next‐generation sequencing

NGS was performed using an amplicon‐based glioma‐tailored gene panel as described previously.^21^ Amplicon sequences were aligned to the human reference genome GRCh37 (hg19) in the target region of the sequence. Data were analyzed using the QIAGEN Web Portal service (https://www.qiagen.com/).

### 2.5 Detection of *EGFR* variants

In all patients, to analyze rearrangement events as significant targets of intragenic CN breakpoints, we sequenced leukocyte DNA for comparison against matched tumor DNA. The *EGFR* variants were detected by reduced read counts obtained for *EGFR* sequences in tumor DNA relative to those in matched leukocyte DNA. In addition, as a validation method, the *EGFR* variants were detected by determining the CN of each exon in *EGFR* and identifying exon sites with −2 SD below in CN compared with the average CN of all exons.

### 2.6 Complementary DNA analyses of clinical specimens

The total RNA from FFPE samples of clinical specimens was extracted using the Maxwell 16 LEV RNA FFPE Purification kit (Promega) and converted into cDNA using the ReverTra Ace qPCR RT Kit & Master Mix (TOYOBO Inc.). The cDNA fragments around the targeted site were amplified by PCR using the KOD One PCR Master Mix (TOYOBO Inc.) using the primers listed in Table S3. The PCR products were analyzed by electrophoresis on 2% agarose gels. The PCR fragments were purified using the Exo‐CIP Rapid PCR Cleanup kit (New England Biolabs Inc.) and analyzed by GENEWIZ Japan Corp.

### 2.7 Immunohistochemistry

Surgical specimens were fixed within 10 min of excision in 10% neutral buffered formaldehyde for 24 h, embedded in paraffin, cut into 3‐μm‐thick sections, and mounted on glass slides coated with poly‐L‐lysine. Subsequently, the sections were probed with 1:25 anti‐EGFR (mouse monoclonal antibody, clone EGFR.113; Leica Biosystems) and 1:200 anti‐EGFRvIII (rabbit monoclonal antibody, cat#64952; Cell Signaling Technology). The sections were then stained with diaminobenzidine tetrahydrochloride and hematoxylin.

### 2.8 Methylation‐specific polymerase chain reaction (MSP)

We performed bisulfite modification of the extracted genomic DNA using the EpiTect Bisulfite Kit (Qiagen). After the conversion, genomic DNA was amplified for the target O^6^‐methylguanine‐DNA methyltransferase promoter (*MGMTp*) region with primers specific to the methylated or unmethylated template using KOD One® PCR Master Mix (TOYOBO). For MSP, two pairs of primers specific for either the methylated or the unmethylated *MGMTp* region were used as previously described.^23^ The amplification was performed with an initial denaturation at 98°C for 1 min and 40 cycles of 98°C for 10 s, 64°C for 5 s. Analysis was performed using the Shimadzu MCE‐202 MultiNA (Shimadzu) on the DNA‐1000 kit.

### 2.9 The MSKCC and KNBTG data analyses

We retrieved the molecular characteristics and clinical information of the GBM cohort from previous publications and performed a validation cohort study using the Memorial Sloan Kettering Cancer Center (MSKCC) database.^24^ After excluding *H3F3A* and *IDH1/2*‐mutant cases, we analyzed 208 patients with *IDH*‐wildtype GBM in MSKCC from the cBioPortal for the Cancer Genomics database (http://cbioportal.org) and analyzed 212 patients with *IDH*‐wildtype GBM from Kansai Molecular Diagnosis Network for CNS tumors (KNBTG).^25^

### 2.10 Definition of actionable alterations

Actionable gene alterations were predicted to confer sensitivity to either an approved targeted agent or an experimental targeted agent currently under clinical trials. The JAX Clinical Knowledgebase (JAX‐CKB) (https://ckb.jax.org/) was used to identify clinically actionable variants.

### 2.11 Data analysis

We used OncoPrinter (cbioportal.org/oncoprinter) and MutationMapper (cbioportal.org/mutation_mapper), which are tools in the cBioPortal for Cancer Genomics, to visualize and analyze our data.^26^, ^27^ Statistical analyses were performed using EZR (Saitama Medical Center, Jichi Medical University). We determined >5 copies as *EGFR* amplification. We compared the patient characteristics with and without *EGFR* amplification and/or mutation using the Chi‐square (*χ* ^2^) test and the Kaplan–Meier log‐rank test, respectively. We also performed univariate and multivariate Cox regression analyses. A difference was considered statistically significant at *p* < 0.05.

## 3 RESULTS

### 3.1 *EGFR* alterations in *IDH* ‐wildtype GBMs

**FIGURE 1:**
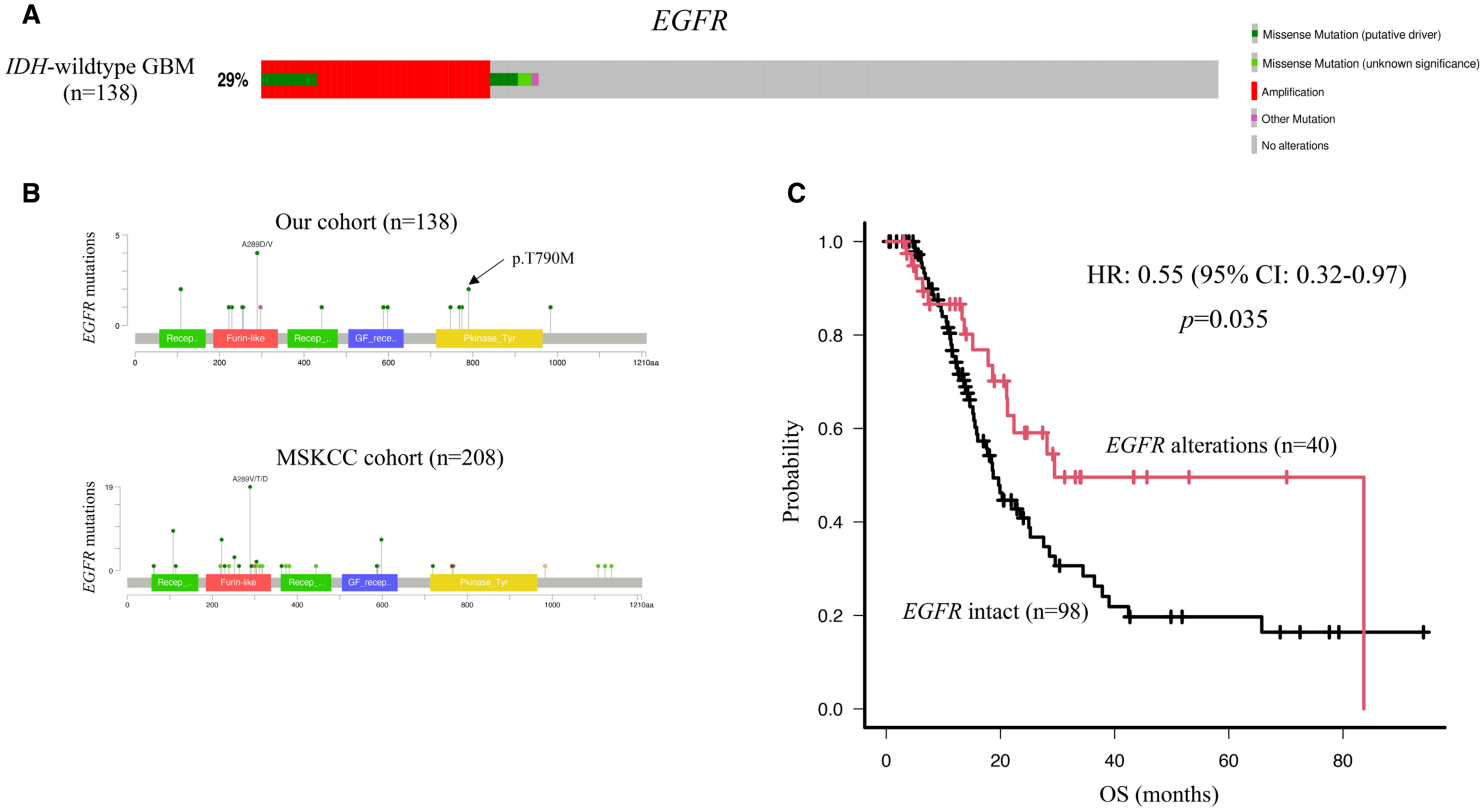
Somatic alterations of *EGFR* and the prognostic impact of *EGFR* status. (A) *EGFR* mutations and copy number alterations were generated and visualized using OncoPrinter included in the cBioPortal for Cancer Genomics software suite. (B) EGFR protein domain structure with somatic mutations summarized from our cohort and MSKCC cohort, respectively. Twenty‐five percent (5/20 mutation sites) *EGFR* mutations in our cohort and 2.1% (3/145 mutation sites) *EGFR* mutations in the MSKCC cohort were kinase domain mutations. (C) *IDH‐*wildtype GBM cases with *EGFR* alterations exhibit significantly longer overall survival than those without *EGFR* alterations. EGFR, epidermal growth factor receptor; IDH, isocitrate dehydrogenase; MSKCC, Memorial Sloan Kettering Cancer Center.

We analyzed 138 *IDH*‐wildtype GBMs. *EGFR* alterations were detected in 29% of *IDH*‐wildtype GBMs (Figure 1A). In detail, 10% of patients with *IDH*‐wildtype GBM had *EGFR* mutations and 23.9% of patients with *IDH*‐wildtype GBM had *EGFR* amplification. Eight cases (5.8%) had both *EGFR* mutation and amplification, seven cases (5.1%) had only *EGFR* mutation, and 25 cases (18.1%) had only *EGFR* amplification. Three cases had two *EGFR* point mutations and one case had three *EGFR* point mutations (Table 1). We also identified mutations in the EGFR extracellular and intracellular domains in 70% (14/20 mutation sites) and 30% (6/20 mutation sites) GBMs, respectively (Figure 1B). Four cases harbored missense mutations in the EGFR kinase domain (encoding L747A, S768I, V774M, and T790M) (Table 1). In GBMs, 25% (5/20 mutation sites) *EGFR* mutations were kinase domain mutations (Figure 1B), and 25% (5/20 mutation sites) *EGFR* mutations were druggable and registered in the JAX‐CKB database (Table 1).

**TABLE 1:** *IDH*‐wildtype GBM cases with *EGFR* alterations

ID	Pathology	Sex	Age	SNV	CNV		Vaf (%)	Impact	Mutation effect	Evidence type
2	GBM	Male	81	p.A289D	19.43	Amplification	80	Missense	Gain of function	−
8	GBM	Female	70	p.S229C	1.76	Intact	28.9	Missense	Gain of function	−
				p.G588D			43.4	Missense	Unknown	−
46	GBM	Female	71	p.A289D	104.10	Amplification	12	Missense	Gain of function	−
50	GBM	Male	67	p.G598V	2.57	Intact	23.6	Missense	Gain of function	−
				p.S768I			19.2	Missense	Gain of function	Druggable mutation
74	GBM	Male	63	p.V774M	29.40	Amplification	75.4	Missense	Unknown	−
				p.T790M			80.5	Missense	Gain of function	Druggable mutation
76	GBM	Male	78	p.L747A	2.48	Intact	19.3	Missense	Unknown	−
81	GBM	Male	39	p.T790M	5.52	Amplification	28.1	Missense	Gain of function	Druggable mutation
84	GBM	Female	65	p.R108K	20.79	Amplification	33.2	Missense	Gain of function	−
				p.A289V			24.17	Missense	Gain of function	Druggable mutation
				p.D984Y			83.37	Missense	Unknown	−
87	GBM	Male	51	p.S442I	2.00	Intact	19.3	Missense	Unknown	−
88	GBM	Male	73	p.F254I	1.98	Intact	29.39	Missense	Unknown	−
118	GBM	Male	61	p.A289V	2.29	Intact	17.4	Missense	Gain of function	Druggable mutation
127	GBM	Male	49	p.R108K	10.49	Amplification	70	Missense	Gain of function	−
128	GBM	Female	80	c.890‐1G>A	1.64	Intact	39.1	Unknown	Unknown	−
135	GBM	Female	64	p.R222C	155.30	Amplification	45.3	Missense	Gain of function	−
137	GBM	Female	70	p.D256V	69.20	Amplification	11.3	Missense	Unknown	−

Abbreviations: CNV, copy number variation; GBM, glioblastoma; SNV, single nucleotide variant; Vaf, variant allele frequency.

### 3.2 Genetic and clinical factors associated with and without *EGFR* alterations

Table 2 compares the genetic and clinical factors of the patients based on their *EGFR* status. We discovered that *TERTp* mutation was more common in GBMs with *EGFR* alterations than in those without (*p* < 0.001). Furthermore, patients with GBM with *EGFR* alterations were associated with higher KPS scores (*p* = 0.014) and lower Ki‐67 scores (*p* = 0.005) than those without *EGFR* alterations.

**TABLE 2:** Background of patients with and without *EGFR* alterations

Prognostic factor	All (*n* = 138)	*EGFR* alterations (*n* = 40)	Intact *EGFR* (*n* = 98)	*p* value
Sex				
Male	79 (57.2%)	22 (55.0%)	57 (58.2%)	0.850
Female	59 (42.8%)	18 (45.0%)	41 (41.8%)	
Age				
<70 years	80 (58.0%)	21 (52.5%)	59 (60.2%)	0.450
≥70 years	58 (42.0%)	19 (47.5%)	39 (39.8%)	
KPS				
≥80 points	63 (45.7%)	25 (62.5%)	38 (38.8%)	0.014*
≤70 points	75 (54.3%)	15 (37.5%)	60 (61.2%)	
Extent of resection				
≥90%	71 (51.4%)	24 (60.0%)	47 (48.0%)	0.260
<90%	67 (48.6%)	16 (40.0%)	51 (52.0%)	
Ki‐67				
≥35%	72 (52.2%)	13 (32.5%)	59 (60.2%)	0.005*
<35%	66 (47.8%)	27 (67.5%)	39 (39.8%)	
*CDKN2A/B* homdel	61 (44.2%)	23 (57.5%)	38 (38.8%)	0.059
*NF1* loss and/or mut	32 (23.2%)	7 (17.5%)	25 (25.5%)	0.378
*PTEN* loss and/or mut	87 (63.0%)	27 (67.5%)	60 (61.2%)	0.562
*TP53* loss and/or mut	63 (45.7%)	15 (37.5%)	48 (49.0%)	0.260
*RB1* loss and/or mut	55 (39.9%)	16 (40.0%)	39 (39.8%)	1.000
*TERTp* mut	90 (65.2%)	35 (87.5%)	55 (56.1%)	<0.001*
Unmethylated *MGMTp*	73 (52.9%)	19 (47.5%)	57 (58.2%)	0.265

Abbreviations: *EGFR*, epidermal growth factor receptor; homdel, homozygous deletion; KPS, Karnofsky Performance Scale; mut, mutation.

* Statistical significance.

### 3.3 *EGFR* alterations are associated with good patient prognoses

We observed a significant difference in the median overall survival (OS) of patients with and without *EGFR* alterations (29.5 and 18.7 months, respectively; *p* = 0.035; Figure 1C). First, we determined whether the identified genetic markers were prognostic markers. Notably, *EGFR* alterations were significant predictors of good prognosis, and unmethylated *MGMTp* was a significant predictor of poor prognosis, as determined by our univariate (HR: 0.55 [0.32–0.97], *p =* 0.038; HR: 2.91 [1.76–4.80], *p <* 0.001; Table S1) and multivariate analyses (HR: 0.38 [0.20–0.72], *p =* 0.003; HR: 3.48 [2.05–5.89], *p <* 0.001; Table S1).

Second, we identified the clinical prognostic factors, which included analysis of the genetic markers for *EGFR* alterations and unmethylated *MGMTp*. Notably, our univariate analysis revealed that higher age (*p* < 0.001), lower KPS scores (*p* = 0.042), extent of resection < 90% (*p* = 0.009), and unmethylated *MGMTp* (*p <* 0.001) were significantly associated with poor prognosis, and *EGFR* alterations (*p* = 0.038) were significantly associated with good prognosis (Table 3). Thereafter, we adjusted the covariates, including sex and age and KPS score and the extent of tumor resection, in the multivariate Cox proportional hazards model. This analysis corroborated that *EGFR* alterations were independent good prognostic markers of OS in patients with *IDH*‐wildtype GBM (*p* = 0.023; Table 3) and unmethylated *MGMTp* was an independent poor prognostic marker of OS in patients with *IDH*‐wildtype GBM (*p <* 0.001; Table 3).

**TABLE 3:** Clinical and genetic prognostic factors

Prognostic factor	Univariate analysis		Multivariate analysis	
	HR (95% CI)	*p* value	HR (95% CI)	*p* value
Sex (male)	1.60 (0.98–2.60)	0.058	1.98 (1.19–3.29)	0.008*
Age (≥70 years)	2.49 (1.54–4.03)	<0.001*	3.04 (1.83–5.07)	<0.001*
KPS score (≤70 points)	1.64 (1.02–2.65)	0.042*	1.46 (0.87–2.45)	0.147
Extent of resection <90%	1.90 (1.18–3.07)	0.009*	1.98 (1.20–3.28)	0.008*
unmethylated *MGMTp*	2.91 (1.76–4.80)	<0.001*	3.86 (2.27–6.55)	<0.001*
*EGFR* alterations	0.55 (0.32–0.97)	0.038*	0.51 (0.28–0.91)	0.023*

Abbreviation: KPS, Karnofsky Performance Scale.

* Statistical significance.

In patients with unmethylated *MGMTp*, *EGFR* alterations were a significant predictor of good prognosis (*p* = 0.048, Figure S1A), whereas in patients with methylated *MGMTp*, there was no significant difference between the median OS of patients with and without *EGFR* alterations (*p* = 0.171, Figure S1B).

### 3.4 Validation cohort

In a validation study, we analyzed the data of 208 patients with *IDH*‐wildtype GBM in the MSKCC and 212 patients with *IDH*‐wildtype GBM in the KNBTG. The frequency of *EGFR* alterations in our cohort (Figure 1A) was lower than that in the MSKCC cohort (29% and 46%, respectively; *p* < 0.001). However, the frequency of *EGFR* mutations in the kinase domain in our cohort was higher than that in the MSKCC cohort (25% and 2.1%, respectively; *p* < 0.001) (Figure 1B). The KNGBT cohort did not include information on *EGFR* mutations.

In the MSKCC cohort, although *EGFR* alterations showed a trend toward better prognosis, there was no statistical significance (*p* = 0.110, Figure S2A). Moreover, in the KNBTG cohort, although *EGFR* amplification showed a trend toward better prognosis, there was no statistical significance (*p* = 0.061, Figure S2B).

### 3.5 *EGFR* variants in *IDH* ‐wildtype GBMs

**FIGURE 2:**
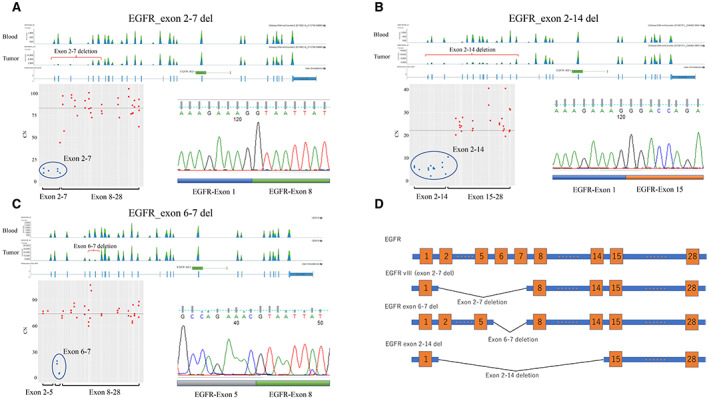
Detection of *EGFR* variants. (A) *EGFRvIII* was indicated by reduced read counts for exons 2–7 in tumor DNA relative to those for exons 2–7 in matched leukocyte DNA (upper panel) and was detected based on reduced CN of exons 2–7 compared with the average CN of all exons (lower left panel). *EGFRvIII* was validated using RT‐PCR (lower right panel). (B) *EGFR* exon 2–14 deletion was indicated by reduced read counts for exons 2–14 in tumor DNA relative to those for exons 2–14 in matched leukocyte DNA (upper panel). It was detected based on reduced CN of exons 2–14 compared with the average CN of all exons (lower left panel). *EGFR* exon 2–14 deletion was validated using RT‐PCR (lower right panel). (C) *EGFR* exon 6–7 deletion was indicated by reduced read counts for exons 6–7 in tumor DNA relative to those for exons 6–7 in matched leukocyte DNA (upper panel). It was detected based on reduced CN of exons 6–7 compared with the average CN of all exons (lower left panel). *EGFR* exon 6–7 deletion was validated using RT‐PCR (lower right panel). (D) Structures of *EGFR* and its splicing variants. *EGFRvIII* (exon 2–7 deletion), exon 6–7 deletion, and exon 2–14 deletion are shown. CN, copy number; *EGFR*, epidermal growth factor receptor.

The *EGFRvIII* (Δ 2–7) variant was detected by NGS in 7 of the 33 tumors with *EGFR* amplification (21.2%) (Table S2). All EGFRvIII‐positive cases were verified by immunohistochemistry and reverse transcription‐polymerase chain reaction (RT‐PCR) (Figure 2A,D). In addition, we identified two other *EGFR* variants in GBM cases, namely deletions of exons 6–7 (Δe 6–7) and exons 2–14 (Δe 2–14). *EGFR* Δe 6–7 was detected in one case, and *EGFR* Δe 2–14 was detected in two cases (Table S2). Both *EGFR* Δe 6–7 and *EGFR* Δe 2–14 were verified by RT‐PCR (Figure 2B–D). All *EGFR* variants showed *EGFR* amplification (Table S2).

### 3.6 Longitudinal and temporal transformation of *EGFRvIII*

**FIGURE 3:**
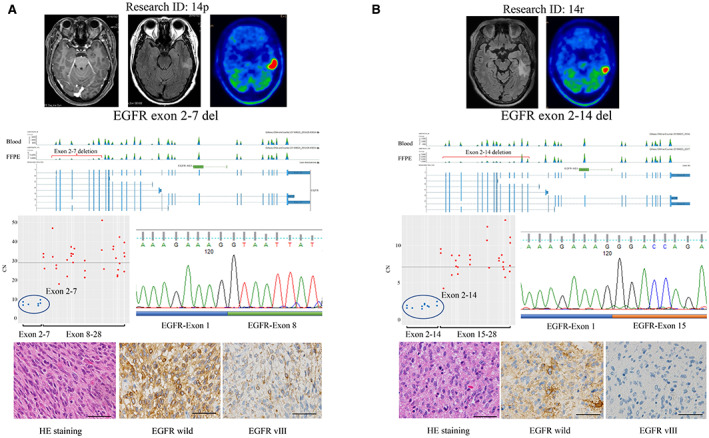
Longitudinal transformation of *EGFRvIII*. (A) Upper: Enhanced T1WI, FLAIR, and methionine PET in an initial GBM case. Middle: *EGFRvIII* detected by NGS and RT‐PCR. Lower: hematoxylin, eosin, and immunostaining of wildtype EGFR and EGFRvIII. (B) Upper: FLAIR and methionine PET in a recurrent GBM case. Middle: *EGFRvIII* detected by NGS and RT‐PCR. Lower: hematoxylin, eosin, and immunostaining for wildtype EGFR and EGFRvIII. EGFR, epidermal growth factor receptor; GBM, glioblastoma; NGS, next‐generation sequencing.

A 57‐year‐old woman with complaints of seizures underwent resection for a lesion in the left temporal lobe with high FLAIR signals observed by magnetic resonance imaging and high methionine accumulation observed by positron emission tomography. The pathological diagnosis was *IDH*‐wildtype GBM with an *EGFRvIII* (Δe 2–7) variant (Figure 3A). Two years later, a tumor recurred in the same area, and the patient underwent another resection. The pathological diagnosis was *IDH*‐wildtype GBM with *EGFR* exon 2–14 deletion (Δe 2–14) without *EGFRvIII* (Δe 2–7) (Figure 3B). In other words, the initial *EGFRvIII* (Δe 2–7) mutation transformed into *EGFR* exon 2–14 deletion (Δe 2–14), with extensive deletion of exons 8–14 at the time of recurrence.

## 4 DISCUSSION

In this study, we collected a higher number of samples than in our previous study.^21^ We found that the frequency of *EGFR* mutations was low but the frequency of EGFR mutations in the kinase domain was high in *IDH*‐wildtype GBMs. Moreover, we developed a method for the detection of *EGFR* transcriptional variants; we found a novel *EGFR* transcriptional variant and identified a case with a rare *EGFR* variant with longitudinal and temporal transformations of *EGFRvIII*.

*EGFR* amplification is detectable in approximately 40%–50% of all GBMs.^1^, ^2^, ^3^ The frequency of *EGFR* alterations in our cohort was lower than that in the MSKCC cohorts. However, studies from Japan reported that 25.5%–33.1% of GBMs have *EGFR* amplification,^25^, ^28^ suggesting that the latter may be less frequent in Japan than in other countries. In addition, lower *EGFR* amplification rates in patients with GBM from Asia were recently reported during a screening for the INTELLANCE1 and INTELLANCE2 randomized GBM trials compared to that in other regions,^29^ which may be due to racial differences. Other reasons may include differences in genetic analysis methods and CN cutoff values. Recent reports indicate that 14.4%–26% GBMs harbor *EGFR* mutations^2^, ^30^; however, the *EGFR* mutation frequency in GBMs in our cohort (10%) was considerably lower than that in the previous study cohort. These results suggest that *EGFR* alterations may be less frequent in Japan than in other countries. Notably, in this study, GBM with *EGFR* alterations had a better outcome than GBM without them. In this validation study, in the MSKCC and KNBTG cohorts, *EGFR* alterations showed a trend toward better prognosis. Consistent with previous reports,^31^, ^32^, ^33^ we also identified the age and extent of surgical resection and unmethylated *MGMTp* as independent GBM prognostic factors, highlighting the accuracy of our study. However, in our study, KPS was not a prognostic factor in the multivariate analysis. The prognostic value of KPS in GBMs has been controversial. Some studies have suggested that KPS is an independent prognostic factor,^32^, ^33^ whereas others showed no associations.^25^, ^28^, ^34^ As for Ki‐67, a previous study has shown that a Ki‐67 staining percentage of >20% predicts poorer survival in *IDH*‐wildtype GBM.^35^ In our study, although we did not examine the prognostic impact of the Ki‐67 score, cases of *IDH* wildtype GBM with *EGFR* alterations were significantly associated with a lower Ki‐67 score.

As for the prognostic value of *EGFR* alterations in GBM, Batchelor et al. reported that *EGFR* amplification was associated with better prognosis in older patients, but worse prognosis in younger patients.^8^ Armocida et al. reported that EGFR expression was not associated with prognostic impact in younger patients and patients older than 45 years.^36^ Such discrepancies between the results of these studies and our study may be due to the differences in the methods used for detecting *EGFR* amplification, the definitions of the extent of resection, and the decision regarding whether biopsy should be excluded. Furthermore, in our study, *EGFR* alterations were good prognosis markers in patients with unmethylated *MGMTp*. Alnahhas et al. reported that GBM with *EGFR* amplification evaluated by NGS had a better outcome in the subgroup of *MGMT* promoter unmethylation,^10^ which was consistent with our results. The key finding of our study is that *EGFR* amplification and/or mutations correlate with better prognostic outcomes. Although other studies suggested the worse prognostic significance of EGFR expression,^11^ EGFR expression was assessed by IHC in most of the studies.

Moreover, in our study, *EGFR* ^*A289D/T/V*^ was the most common missense mutation, and two cases harbored *EGFR* ^*T790M*^ missense mutations in the EGFR kinase domain. Previous studies have shown that approximately 4% of *EGFR* mutations in GBM have mutations in the kinase domain^2^, ^30^; however, 25% of *EGFR* mutations were in the kinase domain in GBMs, and this is higher than that reported previously. Two cases harbored *EGFR* ^*T790M*^ missense mutations in the kinase domain. *EGFR* ^*T790M*^, which is commonly observed in lung cancer,^37^ is a very rare mutation in glioma, with only one case of GBM reported previously.^38^ Osimertinib is an oral, third‐generation tyrosine kinase inhibitor (TKI) that irreversibly inhibits EGFR and was developed specifically to target the *EGFR* ^*T790M*^‐resistant mutation in *EGFR*‐mutated non‐small‐cell lung cancer.^37^, ^39^, ^40^ Makhin et al. reported a GBM case with two *EGFR* point mutations (C628F and A289V) that responded well to osimertinib. Thus, glioma cases with mutations in the EGFR kinase domain may benefit from EGFR TKIs.^41^

*EGFRvIII* is the most common *EGFR* splice variant. *EGFRvIII* activates multiple downstream signaling pathways and exhibits high tumorigenic potential.^14^, ^17^, ^18^ Recent reports indicate that 50%–60% of *EGFR*‐amplified GBMs harbor *EGFRvIII* variants.^15^, ^16^ *EGFRvIII* positivity was detected in 7 of the 33 *EGFR*‐amplified GBMs (21%) in our study, suggesting that *EGFRvIII* positivity may have been less frequent in our study than in previous ones. These discrepancies may be because of ethnic differences in patient cohorts or differences in analysis methods; we used NGS and not IHC or RT‐PCR. A comparative study found that RT‐PCR was more sensitive and specific than immunostaining using two different *EGFRvIII*‐specific antibodies.^42^ Another study showed that the sensitivity of NGS‐based *EGFRvIII* detection is lower than that of immunohistochemistry or RT‐PCR, reflecting that EGFRv*III* may be restricted to small subclones of glioma cells, which may not lead to a detectable reduction in exons 2–7 gene dosage.^43^

In this study, we identified one uncharacterized *EGFR* variant with deletions of exons 6–7 (Δe 6–7). Deletions of exons 2–14 (Δe 2–14) constitute a very rare variant in glioma, with only one case of GBM having been reported previously.^44^ In one case, the initial *EGFRvIII* (Δe 2–7) mutation transformed into an *EGFR* exon 2–14 deletion (Δe 2–14) at the time of recurrence. Recent reports indicate that 16%–59% of GBMs that were initially *EGFRvIII* positive, lost *EGFRvIII* at recurrence.^15^, ^16^, ^20^ Some reports suggest that the frequency of *EGFRvIII* loss at recurrence is altered by the treatment received,^45^ whereas others suggest that it is not dependent on the treatment received.^20^ In addition, it has been reported that the expression of *EGFRvIII* is a result of epigenetic regulation,^46^ but the mechanism by which *EGFRvIII* is lost at recurrence is unknown. Our findings suggest that initial *EGFRvIII* mutations may have been transformed to other variants during recurrence. The functional significance of the novel *EGFR* variants needs to be analyzed in further studies.

In conclusion, we report the distribution of mutations within the *EGFR* coding sequence and two novel *EGFR* variants, one of which showed the longitudinal and temporal transformation of *EGFRvIII*. In addition, we showed that the frequency of driver gene alterations in GBMs differs across cohorts and *EGFR* alterations are an independent predictor of good prognosis. Thus, to implement personalized medicine, it is necessary to accurately assess the genetic profile of each cohort.

## AUTHOR CONTRIBUTIONS

Conception and design: Akihide Tanimoto, Koji Yoshimoto; development of methodology: Toshiaki Akahane, Taiji Hamada, Seiya Yokoyama, Tomoko Takajo, Kei Matsuo; acquisition of data: Nayuta Higa, Hajime Yonezawa, Hiroyuki Uchida, Mari Kirishima, Ryutaro Makino, Shoji Watanabe, Shingo Fujio; analysis and interpretation of data: Nayuta Higa, Toshiaki Akahane, Taiji Hamada, Seiya Yokoyama; manuscript writing, review, and/or revision: Nayuta Higa, Ryosuke Hanaya, Akihide Tanimoto, Koji Yoshimoto; administrative, technical, or material support (e.g., reporting or organizing data and constructing databases): Nayuta Higa, Tomoko Takajo, Hajime Yonezawa, Hiroyuki Uchida, Toshiaki Akahane, Mari Kirishima, Taiji Hamada, Seiya Yokoyama, Kei Matsuo; study supervision: Ryosuke Hanaya, Akihide Tanimoto, Koji Yoshimoto.

## FUNDING INFORMATION

The authors received no financial support related to this study.

## CONFLICT OF INTEREST

The authors declare that they have no conflict of interest.

## ETHICS APPROVAL

The study was approved by the Institutional Review Board of Kagoshima University (approval number: 180104) and complied with the tenets of the Declaration of Helsinki.

## PATIENT CONSENT

Informed consent was obtained from all patients.

## Supporting information


Figure S1

Figure S2

Table S1

Table S2

Table S3

## Data Availability

All data used and analyzed in the current study are available from the corresponding author upon reasonable request. The data are not publicly available due to privacy or ethical restrictions.
