# Supplementary figures and images for: Distribution and favorable prognostic implication of genomic EGFR alterations in IDH ‐wildtype glioblastoma
Source: Cancer Med. 2022 Jun 13;12(1):49–60. doi: 10.1002/cam4.4939 (PMC9844636; doi:10.1002/cam4.4939)

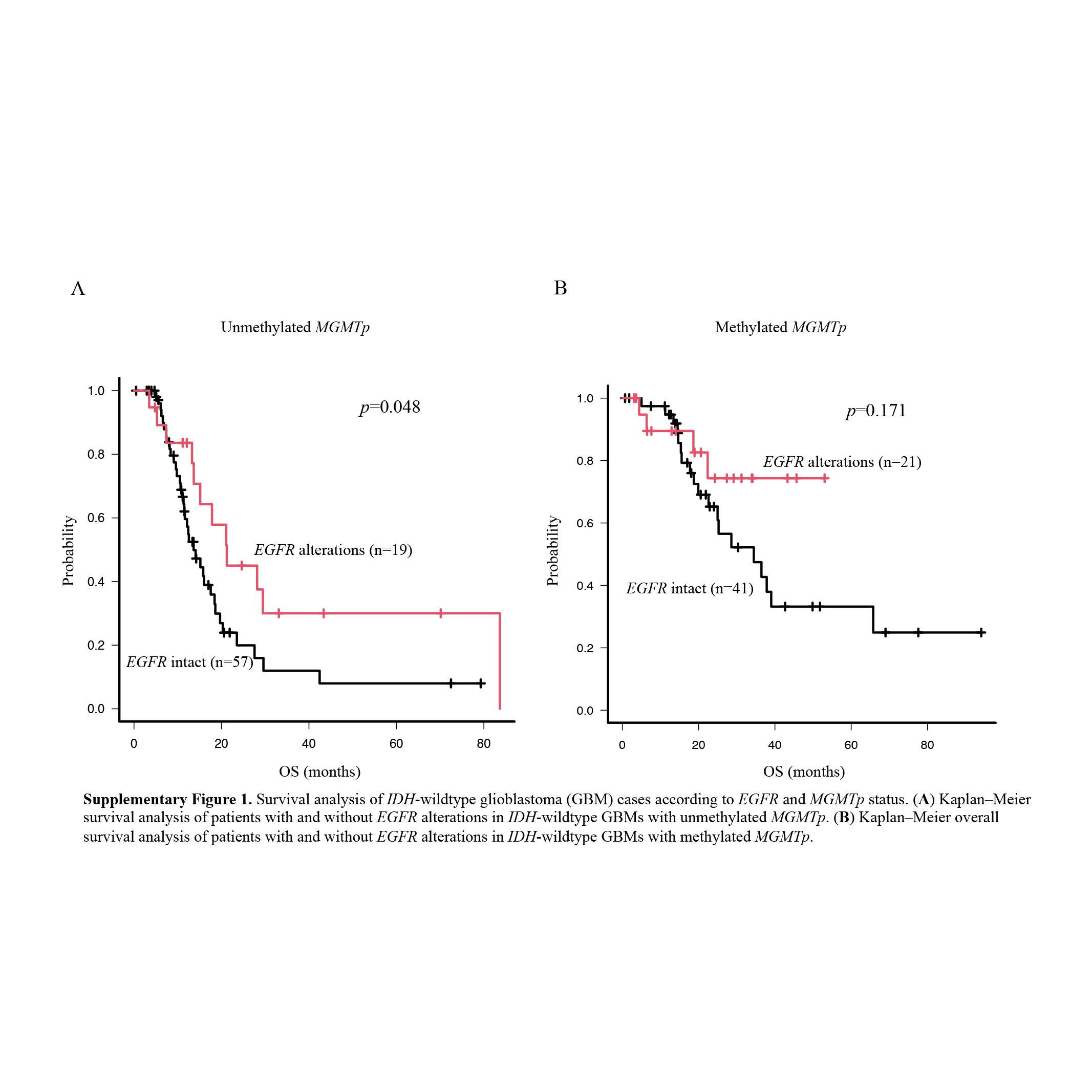

Supplement: Supplementary file 1 — Figure S1 [file CAM4-12-49-s005.jpg]

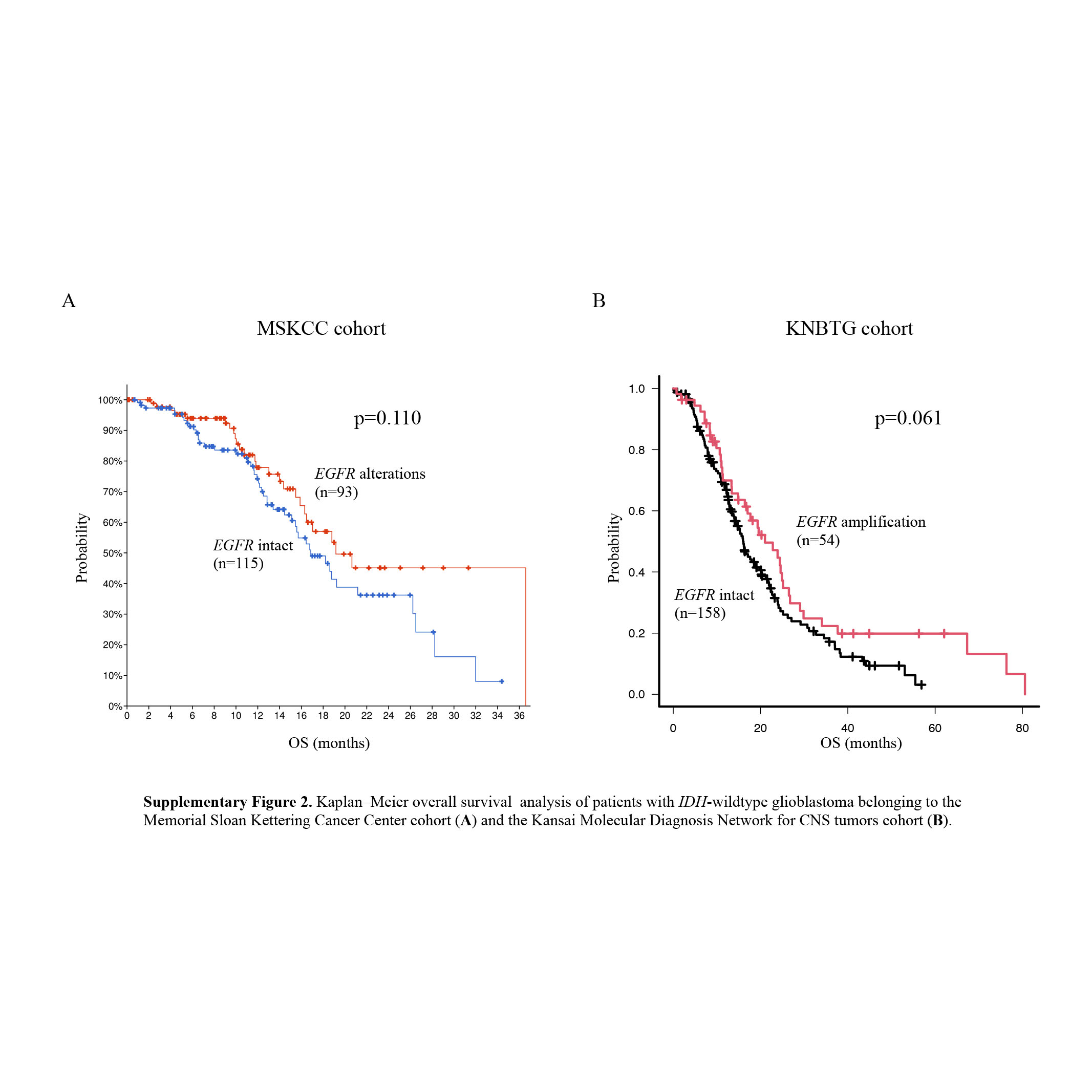

Supplement: Supplementary file 2 — Figure S2 [file CAM4-12-49-s003.jpg]
