# Supplementary material for: Distribution and favorable prognostic implication of genomic EGFR alterations in IDH ‐wildtype glioblastoma
Source: Cancer Med. 2022 Jun 13;12(1):49–60. doi: 10.1002/cam4.4939 (PMC9844636; doi:10.1002/cam4.4939)
Supplement: Supplementary file 3 — Table S1 [file CAM4-12-49-s004.docx]

Supplementary table 1. Genetic prognostic factors

|  | Univariate analysis | | Multivariate analysis | |
| --- | --- | --- | --- | --- |
| Genetic marker | HR (95% CI) | *p*-value | HR (95% CI) | *p*-value |
| *CDKN2A/B* homdel | 1.26 (0.79-2.02) | 0.326 | 1.69 (1.03-2.79) | 0.039* |
| *NF1* alterations | 0.74 (0.42-1.30) | 0.293 | 0.87 (0.48-1.55) | 0.625 |
| *RB1* alterations | 0.87 (0.54-1.40) | 0.565 | 0.88 (0.53-1.48) | 0.635 |
| *PTEN* alterations | 0.64 (0.39-1.07) | 0.087 | 0.54 (0.30-0.99) | 0.049* |
| *TP53* alterations | 1.26 (0.79-2.02) | 0.335 | 1.20 (0.73-1.99) | 0.470 |
| *TERTp* mut | 0.999 (0.62-1.62) | 0.995 | 1.21 (0.67-2.17) | 0.525 |
| unmethylated *MGMTp* | 2.91 (1.76-4.80) | <0.001* | 3.48 (2.05-5.89) | <0.001* |
| *EGFR* alterations | 0.55 (0.32-0.97) | 0.038* | 0.38 (0.20-0.72) | 0.003* |

mut, mutation; homdel, homozygous deletion

*indicates statistical significance.
